# The Anatomy of the Global Football Player Transfer Network: Club Functionalities versus Network Properties

**DOI:** 10.1371/journal.pone.0156504

**Published:** 2016-06-02

**Authors:** Xiao Fan Liu, Yu-Liang Liu, Xin-Hang Lu, Qi-Xuan Wang, Tong-Xing Wang

**Affiliations:** 1 School of Computer Science and Engineering, Southeast University, Nanjing, China; 2 Key Laboratory of Computer Network and Information Integration, Southeast University, Ministry of Education, Nanjing, China; Tianjin University, CHINA

## Abstract

Professional association football is a game of talent. The success of a professional club hinges largely on its ability of assembling the best team. Building on a dataset of player transfer records among more than 400 clubs in 24 world-wide top class leagues from 2011 to 2015, this study aims to relate a club’s success to its activities in the player transfer market from a network perspective. We confirm that modern professional football is indeed a money game, in which larger investment spent on the acquisition of talented players generally yields better team performance. However, further investigation shows that professional football clubs can actually play different strategies in surviving or even excelling this game, and the success of strategies is strongly associated to their network properties in the football player transfer network.

## Introduction

Association football, also referred to as soccer, is probably the sport that gained most global popularity since the 20th century. Professional football industry has thereby grown into a prosperous playground for wealthy investors and big name companies. It was reported that the combined revenue of the top 20 earning clubs in season 2014/15 was over 6.6 billion euros [1]. Meanwhile, the abundant football statistics gathered have attracted fans and scholars to analyze this fascinating game in a quantitative way [2]. Especially, the success of a professional club or a football team is found to be linked to the club investment [1], social network effects [3], the coordination and interaction among team members in matches [4] or even the color of shirts a team wears on the pitch [5]. However, the achievement of a club is ultimately decided by a squad of 30 players who play the matches through the entire season. Therefore, it is eventually the club’s ability of gathering the most talented players which decides about the outcome of the billion euros’ investments.

Actually, talent is believed to be the most important asset in any organization. From senior executives in public companies to common laborers in a massive amount, the acquisition of talents is found to be significantly related to the accomplishment of companies or even national economics [6, 7]. A professional football club usually acquires players from other clubs either permanently, i.e., transfer, or temporarily, i.e., loan, by exchanging certain compensation, i.e., transfer fee. The football player movement has already drawn attention from the academia since decades [8, 9]. However, despite using the football player transfer market as a resource of understanding labor mobility and globalization [10–12], few studies have addressed the relationship between the activities in the transfer market and the success of a club in a systematic way. In this paper, we try to solve this problem by employing a network perspective analysis to the global football player transfer market.

Many natural and man-made systems composed of connected components can be modeled by networks, whose properties are proven to be associated with the functionalities of the systems or their components. Scale-free structure is a common macroscopic property found in social networks. It has been shown that this property could be the cause of the unpredictability of epidemic spreading [13]. Degree-degree mixing is another macroscopic measure which affects the ability of an evolving system to achieve consensus [14]. Microscopic network properties refer to the topological properties of individual nodes and edges and are widely used to measure the functionalities of system components. They were used successfully to characterize the importance of web pages [15], to identify influential spreaders in social networks [16], to find drug targets [17], to characterize dynamic behavior in gas-liquid two-phase flow [18, 19] and to explain the roles taken by research institutions in applying for science funding [20].

Building on a dataset of transfer records from 2011 to 2015 of 410 professional clubs in 24 world-wide top class leagues, our work analyzes the properties of the global football player transfer network at both macroscopic and microscopic scales. In this network, nodes are the elite clubs, and the directed edges connecting the nodes are the player transfers and loans. Particularly, the relationship between node properties and the functionalities of professional clubs is studied. Our results show that clubs’ match performance and profitability from the transfer market are strongly associated with the coreness and brokerage properties of their corresponding nodes in the player transfer network.

## Results

### An overview of the football player transfer market

**Fig 1:**
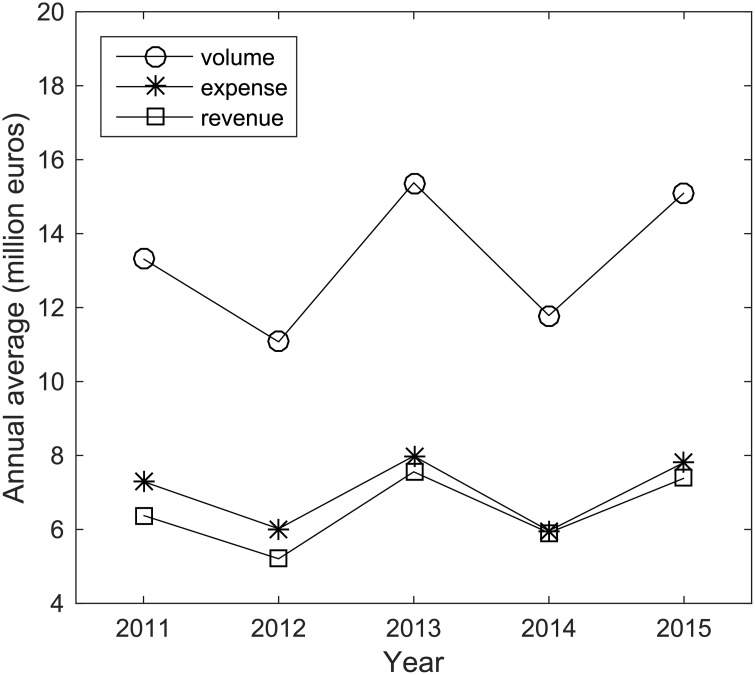
Trend of spending in the global football player transfer market. The trend of average annual expense, revenue and volume (sum of expense and revenue) of the elite clubs in the transfer market.

Professional football is famous for its scarcity of talents. Therefore, wealthy clubs are willing to pay millions of euros in exchange for one qualified player. Moreover, the average annual expense, revenue and volume, i.e., the sum of expense and revenue as a measure of the flow of transfer fees through a club, of clubs show an increasing trend from 2011 to 2015 (Fig 1). The top 10 clubs in terms of annual expense, revenue and volume are summarized in Table A in S1 File. The average expense of the clubs was over 5 million euros and the average transfer fee volume exceeded 10 million euros.

**Fig 2:**
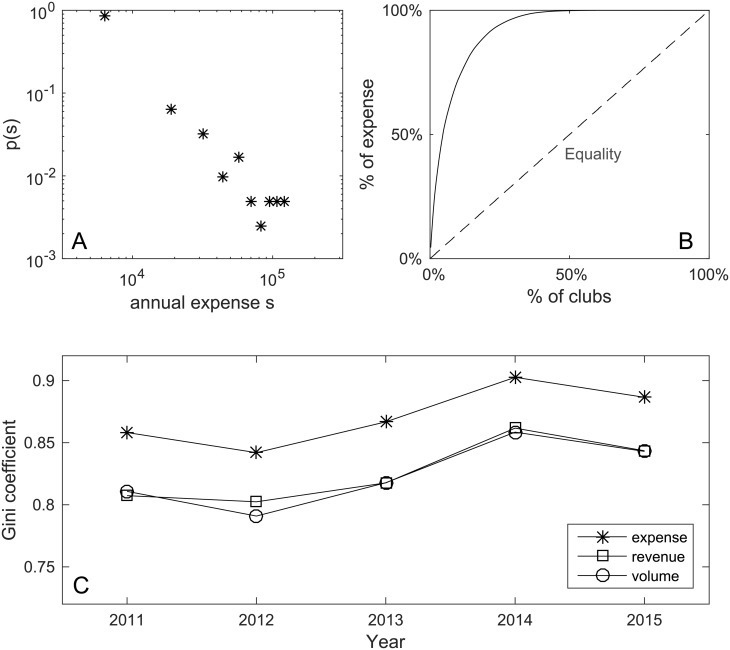
Inequality of financial ability of professional clubs. A: Distribution of annual club expenses in euros. B: Cumulative distribution of annual club expenses. Clubs are sorted in descending order by the transfer expense, and the percentage of total expense was plotted against the corresponding percentage of clubs. The dashed line denotes the situation where all clubs spend the same amount of money in the player transfer market. C: Gini coefficient of annual transfer expense, revenue and volume of the clubs.

However, the financial resources of the elite clubs are not evenly distributed. Fig 2A shows the distribution of average annual expenses of the clubs in 5 years. The distribution is heavy-tailed, in which top spending clubs have the ability of raising an amount of money 10 times larger than most of the clubs. Fig 2B shows the proportion of clubs and their cumulative expenses in the transfer market in a percent scale. It is shown that 80% of the total transfer fee is spent by less than 20% of the clubs. Fig 2C shows the Gini coefficient [21] of transfer expenses of all clubs from 2011 to 2015. An increasing trend indicates that the inequality in financial ability of professional football clubs is gradually magnifying. Overall, we have observed substantial and increasing inequality in the financial ability of professional clubs.

### Construction of the global football player transfer network

**Fig 3:**
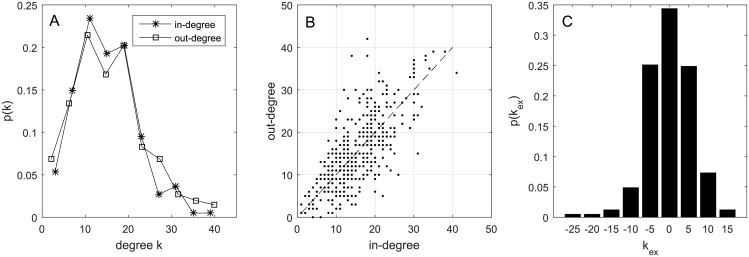
Degree distributions of the global football player transfer network. A: The in-degree and out-degree distributions of the network. B: The in-degree/out-degree relation for each club in the transfer network. C: The distribution of excess degree *k*_ex_ of all nodes in the transfer network. The standard deviation of *k*_ex_ is 5.7.

In the 5 year period from 2011 to 2015, there were totally 8948 transfer actions, excluding loans, among the elite clubs, constituting a player transfer network of 410 nodes connected by 6316 directed edges, where the direction of edges denotes the direction of player movements. All the nodes in the network are strongly connected except for three nodes with only outgoing edges and no incoming edges. The mean shortest distance between all nodes is 2.8 and the clustering coefficient is 0.21. Here, the calculation of the mean shortest distance and the clustering coefficient both take the directions of the edges into account. For the clustering coefficient, we have adopted the simple definition proposed in [22] that it is the average ratio between all directed triangles actually formed by each node and the number of all possible triangles that the node could form. Comparing to the average mean shortest distance of 2.5 and the average clustering coefficient of 0.04 in random networks of the same size and connection density, the player transfer network exhibits the small-world phenomenon [23]. Fig 3A shows the distributions of in-degree *k*_in_ and out-degree *k*_out_ of the network. The distributions are highly skewed but not as unequal as that of the transfer expense in Fig 2A. Fig 3B and 3C show the correlation of in-degree and out-degree and the distribution of excess degree *k*_ex_ = *k*_in_ − *k*_out_ of all nodes respectively. It is shown that the numbers of clubs that a professional club “buys from” and “sells to” are basically equal.

**Fig 4:**
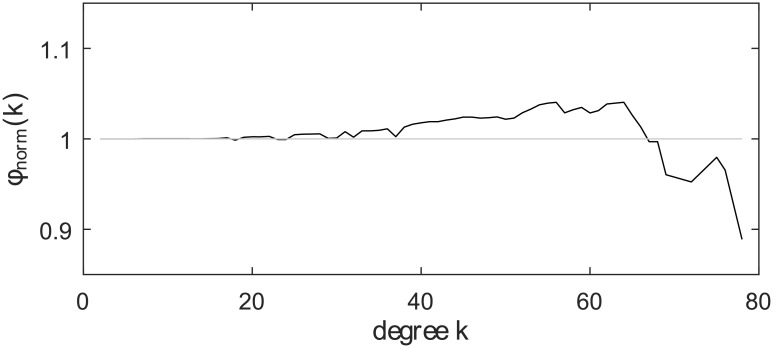
Rich-club coefficients in the player transfer network. The network fails to show the rich-club phenomenon *ϕ*_norm_(*k*) > 1 for high degree nodes.

The rich-club phenomenon refers to the tendency that well-connected nodes also connect to each other. This phenomenon is commonly found in social networks and transportation networks [24]. To evaluate this phenomenon in the player transfer network, we computed the rich-club coefficient, *ϕ*_norm_(*k*), for all node degrees *k* (see Data and Methods for detailed information on this measure). A value *ϕ*_norm_(*k*) > 1 for large *k* indicates that high degree nodes are organized as a rich club. Fig 4 shows that the rich-club phenomenon actually cannot be observed for the clubs with the highest degrees. On the contrary, it suggests that the clubs which exchange players with the largest number of peer clubs tend not to exchange players among themselves and that the global transfer market may have disparate “cores” that control compartmented transfer resources.

### Club functionalities versus network properties in the transfer network

**Fig 5:**
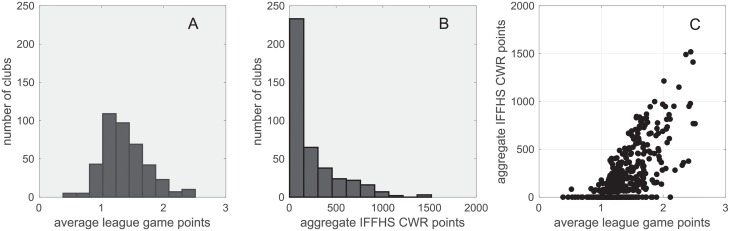
Match performance of the elite clubs. A: The distribution of average league game points. B: The distribution of 5 year aggregate IFFHS CWR points. C: Scatter plot of the two match performance measures.

In this section, we will explore the relationship between the functionalities of a club and its network properties. The ultimate measure of the success of a commercial organization is its profitability, which also applies to a professional football club. Generally speaking, clubs with the highest achievements in prestigious competitions are also the ones that generate the largest revenue from various commercial activities [1]. Meanwhile, a club could also profit directly from the transfer market, by receiving more compensation from the players transferred out than it pays to acquire new players. Therefore, the club functionalities can be described either by its match performance or its transfer profit. Match performance includes the domestic and international match results. We quantify domestic performance of a club by the average game points in its domestic league matches from 2011 to 2015. On the other hand, the five year aggregate IFFHS Club World Ranking (CWR) point is employed to quantify the overall performance of a club in both domestic and international competition [25]. Table B in S1 File shows the top 10 clubs in terms of their match performance. Fig 5 shows that although the distributions of average league game points and aggregate IFFHS CWR points exhibit different characteristics, the two performance measures are positively correlated. The ability of profiting from player transfers are defined by two measures, i.e., the average annual transfer balance and the cumulative price overflow from player transits. If a player has transferred from club A to club B then to club C, we define that the player has transited through club B. The price overflow of this player in club B is the difference between the transfer fees payed by club C to club B and by club B to club A. Table C in S1 File shows the top 10 clubs in terms of profitability in the transfer market. Table 1 shows that the two categories of club functionalities are generally weakly or not correlated. Overall, match performance and transfer profitability can be considered as independent indicators of the functionalities of a professional football club.

**Table 1:** Kendall’s Tau between match performance and profitability of clubs.

	Avg. league pts.	Agg. CWR pts.
Balance	0.08	0.16
Price overflow	0.01	0.00

Which factors of the transfer market affect the functionalities of professional football clubs? To answer this question, we first examine the relationship between the amount of money a club has spent at the transfer market and its functionalities. Table 2 shows Kendall’s Tau coefficients [26] between the average annual transfer fee quantities, i.e., average annual revenue, expense and total volume, and the functionality measures of the clubs. It is shown that the amount of transfer fee is positively correlated with club performance, which indicates that the more money a club spends or receives at the transfer market, the better match performance it will generally achieve. In addition, this “money effect” is more obvious in the clubs’ international performance than in their domestic performance. Meanwhile, Table 2 also shows that the expense and transfer fee volume are basically negatively correlated with the profitability from player transfers of a club. This indicates that for most clubs, their transfer expenses are larger than the transfer revenue.

**Table 2:** Kendall’s Tau between the money flow of clubs at the transfer market and their match performance.

	Avg. league pts.	Agg. CWR pts.	Balance	Price overflow
Volume	0.35	0.54	-0.52	0.17
Revenue	0.34	0.53	-0.14	0.37
Expense	0.32	0.49	-0.73	0.01

Next, we study the relationship between the functionalities of the clubs and their properties in the player transfer network. The node properties of concern include two categories of metrics that measure the coreness and brokerage of the nodes, respectively. The coreness of a node measures the richness of its connections and is usually described by the number of direct connections combined with the number of indirect connections [27]. In this paper, eigenvector centrality and PageRank centrality are chosen as the coreness metrics. The coreness metrics could indicate the search breadth of the club scouts in the transfer market. Table D in S1 File shows the top 10 clubs in terms of coreness properties in the player transfer network. The brokerage of a node measures the extent to which it controls the network flow [20]. Clubs with large brokerage metrics tend to exclusively control certain transfer resources and act as brokers amongst other clubs. In this paper, effective size, closeness centrality and betweenness centrality are chosen as the brokerage metrics. Table E in S1 File shows the top 10 clubs in terms of brokerage properties in the player transfer network. Table 3 shows Kendall’s Tau between club functionalities and network properties. The match performance, especially international performance, of the clubs is positively correlated with the brokerage metrics, while clubs’ profitability from transfers is generally weakly or not correlated with the network properties. This result suggests that the brokerage power of a club plays the most significant role in determining its match outcome, while the relationships between other network measures and club functionalities are relatively weak. In the next sections, we will further explore the relationships between club functionalities and node properties from several different aspects.

**Table 3:** Kendall’s Tau between network properties and club functionalities in the player transfer network.

	Eigenvector centrality	PageRank centrality	Effective size	Betweenness centrality	Closeness centrality
Avg. league pts.	0.18	0.12	**0.34**	**0.34**	**0.33**
Agg. CWR pts.	0.36	0.14	**0.48**	**0.37**	**0.49**
Balance	0.04	-0.02	0.19	0.16	0.26
Price overflow	0.04	0.04	0.06	0.06	0.07

### Club functionalities versus network properties in international and domestic transfer networks

International sports labor migration shows characteristics different from those of domestic sports labor movements [11, 12]. In this paper, the football player transfer network can also be separated into two subnetworks accordingly. The domestic transfer network contains only transfers within a same league and the international transfer network contains only international transfers between different leagues. Table 4 shows the correlation of network properties and club functionalities in both international and domestic transfer networks. It is shown that the node properties taking account of global network connections, i.e., eigenvector centrality, PageRank centrality, betweenness centrality and closeness centrality, are better indicators of both domestic and international match performance, while the node property taking account of local network connections, i.e., effective size, in the domestic transfer network is a better indicator of match performance.

**Table 4:** Kendall’s Tau between network properties and club functionalities in international and domestic transfer networks.

		Eigenvector centrality	PageRank centrality	Effective size	Betweenness centrality	Closeness centrality
Avg. league pts.	International	**0.29**	**0.19**	0.03	**0.28**	**0.36**
	Domestic	-0.04	-0.06	**0.34**	0.04	0.15
Agg. CWR pts.	International	**0.40**	**0.24**	0.12	**0.36**	**0.48**
	Domestic	0.13	-0.05	**0.45**	0.10	0.23
Balance	International	0.05	0.04	0.11	0.15	0.23
	Domestic	0.07	0.04	0.16	0.10	0.08
Price overflow	International	0.03	0.03	0.01	0.07	0.07
	Domestic	0.02	0.04	0.06	0.01	-0.01

### Club functionalities versus node properties in transfer network and loan network

The movement of a player can take two different forms, i.e., transfer and loan. In a transfer, the player terminates his contract with the former club and signs a contract with the new club, while in a loan, the player is allowed to temporarily play for a club other than the one he is currently contracted to. The player loan network can be constructed similarly to the player transfer network, in which the nodes are clubs and directed edges are player loans. However, loan network and transfer network show different characteristics. In the 24 leagues, only 394 out of 410 clubs are involved in player loans. The number of edges is 2509, with totally 3586 player loan records. All the nodes in the loan network are weakly connected but the network has 129 strongly connected components. The clustering coefficient in the loan network is 0.11 compared to 0.21 in transfer network. The average path length is 4.1, compared to 2.8 in the transfer network. Therefore, the loan network is a sparsely and weakly connected network with multiple weakly disconnected subnetworks. Moreover, the properties of nodes in the two networks are loosely related. Although the correlations of eigenvector centrality, effective size and betweenness centrality of the same nodes in the two networks are positive and strong (0.46, 0.75 and 0.51, respectively), those of the PageRank centrality and closeness centrality of the same nodes in both networks are weak (-0.02 and 0.08, respectively).

The correlations among network properties and club functionalities in transfer network and loan network are shown in Table 5. Similar to the transfer network, the node brokerage properties are found to be strongly related to match performance. However, the coreness properties in the loan network no longer have positive correlations with the match performance of the club. Actually, the differences between the correlations of coreness properties and the match performances in loan and transfer networks are quite large. The reason is that instead of players loaned-in, players loaned-out indicate the excessiveness of player resources of a club and hence the eigenvector centrality and the PageRank centrality actually cannot capture the attraction of clubs to players as they do in player transfer network. Nonetheless, our finding shows that the larger and more exclusive the player resources one club controls, the better overall performance it will achieve.

**Table 5:** Kendall’s Tau between network properties and club functionalities in loan and transfer networks.

		Eigenvector centrality	PageRank centrality	Effective size	Betweenness centrality	Closeness centrality
Avg. league pts.	Loan	**-0.07**	**-0.20**	0.22	0.28	0.23
	Transfer	**0.18**	**0.12**	0.34	0.34	0.33
Agg. CWR pts.	Loan	**0.11**	**-0.12**	0.43	0.39	0.33
	Transfer	**0.36**	**0.14**	0.48	0.37	0.49
Balance	Loan	0.10	0.01	0.13	0.12	0.06
	Transfer	0.04	-0.02	0.19	0.16	0.26
Price overflow	Loan	0.12	0.14	0.05	0.05	-0.02
	Transfer	0.04	0.04	0.06	0.06	0.07

### Club functionalities versus node properties in money leagues and farm leagues

Table 1 has shown that the match performance and profitability of clubs are weakly or not related. However, a closer examination shows that the clubs in different leagues exhibit different characteristics in the relationship between the two kinds of club functionalities. In some leagues, the more transfer profit a club generates, the better match performance it achieves while in other leagues it is just the opposite (S1 Fig). We roughly classify the leagues into three categories, i.e., the leagues with negative correlation between the clubs’ annual balance and average league game points with adjusted *R*^2^ > 0.25 are referred to as “money leagues”, as the more the clubs spend, the better match performance they achieve; the leagues with positive correlation and adjusted *R*^2^ > 0.25 are referred to as “farm leagues”, as the more the clubs profit from the transfer market, the better match performance they achieve; the rest are referred to as “outlier leagues”. In money leagues, the clubs’ performances are strongly related to their abilities in raising transfer fund. In farm leagues, the club’s performances are strongly related to their abilities of profiting from player transfer. In the outlier leagues, there is little interdependence between match results and clubs’ profitability from player transfer. A summary of our classification is shown in Table 6.

**Table 6:** Classification of the 24 leagues according to the relationship between match performance and transfer profitability of their teams.

Money leagues	England, France, Germany, Italy, Russia, Spain, Turkey, Ukraine
Farm leagues	Argentina, Belgium, Brazil, Chile, Finland, Greece, Netherlands, Norway, Portugal, Romania, Scotland, Sweden
Outlier leagues	Australia, Ireland, Mexico, USA

The relationship between the node properties in the player transfer network and the functionality of clubs in different league categories is shown in Table 7. The parameter correlations of clubs in “money leagues” generally agree with the average nodes in the transfer network. However, in “farm leagues”, the correlation between eigenvector centrality, effective size, closeness centrality and club domestic match performance vanishes, but strong correlation emerges between node coreness and brokerage and club profitability. Especially, the brokerage of a club in the player network is strongly correlated to its annual balance. This phenomenon suggests that clubs in different financial environments actually have various strategies of achieving success, either by acquiring the best players at all costs, or by cultivating players with potential and profit from the reselling of these valuable assets. More importantly, the successes of both strategies are strongly related to the clubs’ network properties in the global player transfer network.

**Table 7:** Kendall’s Tau between network properties and club functionalities in different league categories.

		Eigenvector centrality	PageRank centrality	Effective size	Betweenness centrality	Closeness centrality
Avg. league pts.	Money Leagues	**0.25**	**0.15**	**0.33**	**0.29**	**0.34**
	Farm Leagues	**-0.01**	**0.03**	**0.06**	**0.15**	**0.04**
	Outlier Leagues	0.18	0.11	0.41	0.43	0.43
Agg. CWR pts.	Money Leagues	0.30	0.08	0.44	0.39	0.51
	Farm Leagues	0.43	0.25	0.53	0.41	0.51
	Outlier Leagues	0.12	-0.04	0.16	0.07	0.17
Balance	Money Leagues	-0.13	-0.14	-0.09	-0.09	-0.03
	Farm Leagues	**0.35**	**0.20**	**0.56**	**0.46**	**0.59**
	Outlier Leagues	-0.21	-0.19	-0.14	-0.05	0.01
Price overflow	Money Leagues	0.03	-0.01	-0.04	-0.05	-0.03
	Farm Leagues	**0.19**	**0.14**	**0.23**	**0.17**	**0.23**
	Outlier Leagues	-0.06	-0.02	-0.08	-0.02	-0.03

## Discussion

Football is probably the most popular sports in the world. The abundance of statistics regarding teams’ activities on and off the pitch has attracted extensive quantitative analysis by fans and scholars from various perspectives. However, despite the importance of the acquisition of talented players to the success of a professional football club, previous studies rarely addressed the relationship of the clubs’ functionality to their activities in the player transfer market. To do so, we have collected exhaustive transfer records among more than 400 football clubs in major professional leagues from different countries during the last 5 years. Data reveals that football is indeed a money game, in which clubs spend large amounts of money on football stars in order to achieve prestigious status and generate commercial revenue. However, in this winner-takes-all game, the financial abilities of clubs are severely unequal. Wealthy clubs with overwhelming financial resources could spend tens or even thousands times more money than normal clubs on acquiring better players, therefore other clubs must seek different strategies to survive in this competitive industry.

Network science provides a systematic perspective and a variety of tools to quantitatively study the structure of complex systems. Particularly, the network properties of system components are found closely related to their functionalities. In this work, we have employed a network perspective to analyzing the global football player transfer market. In the transfer network, nodes are clubs linked by directed edges representing player transfers. The global football player transfer network is a small-world network with multiple loosely connected hubs. Clubs that act as hubs or brokers in the network usually achieve better domestic and international match performances. Football players contracted to a club can also move to other clubs on loan terms. A player loan network can be constructed similarly to the player transfer network. In the player loan network, clubs acting as brokers are generally the ones that achieve better match performance. The results suggest that professional clubs should develop their scouting abilities and maintain exclusive player resources in order to achieve better match performances.

The ultimate goal of commercial organizations is to make profit. However, depending on various factors, football industry does not generate a comparable amount of revenue across the world. In some leagues, clubs could spend millions of euros on building a better team and profit from commercial activities such as selling TV broadcasting rights or portraiture rights. Yet, this strategy might not apply to clubs in leagues that attract less financial attention. Therefore, cultivating players with high potential and selling them to wealthier leagues is another viable way of generating profit for the clubs. No matter which strategy a club has to choose, in order to achieve success, the club must carefully select its position, particularly the coreness and brokerage properties, in the global player transfer network.

Considering the international and domestic nature of player transfers and loans, the global player transfer network is actually an ensemble of multiple layers of networks, or a network of networks. How the relationships and interaction mechanisms among different layers or subnetworks can be linked to the formation of the global player transfer network and to the functionalities of its components are worth further investigation.

Meanwhile, the domestic and international movement of football players is merely a special case of the ongoing urbanization processes and global labor migration today. How the acquisition and loss of labors with different skill sets could affect the economic status of regions or nations is still an open question. We believe that the systematic perspective and network-based methods employed in this work can be further extended to study this question with a promising outcome.

## Data and Methods

### Transfer records

The transfer and loan records in and out of 410 clubs in 24 top-class professional football leagues (Table 6) across the world from 2011 to 2015 are retrieved from the online football database Soccerway (whose data provider is Opta Sports). The dataset contains 31128 transfer records and 13837 loan records, with totally 3660 clubs and 23765 players involved. Transfers and loans are recorded in various forms: transfer with an explicit transfer fee, free transfer, transfer with a undisclosed fee, swap and loan. Transfers with undisclosed fee and player swaps are treated as free transfers in this paper.

### Match performance

Each win in the domestic league game is counted 3 points, each draw 1 point and each lose 0 points. The IFFHS Club World Ranking points are composed by the International Federation of Football History & Statistics (IFFHS) based on a set of rules composing both domestic and international match results [25]. The CWR points are updated once per month, the aggregate CWR points used in this paper covers the period from January 2011 to December 2015.

### Rich-club coefficient

The rich-club coefficient *ϕ*_norm_(*k*) is a measure of connection density of high degree nodes in the network [24]. First, we calculate the value *ϕ*(*k*) = 2*E*(*k*)/*k*(*k* − 1), where *E*(*k*) is the number of edges among nodes with a degree ≥ *k*. Then we determine *ϕ*_rand_(*k*) as the average value in 1000 random graphs with the same node degrees as in the empirical network. Finally, *ϕ*_norm_(*k*) = *ϕ*(*k*)/*ϕ*_rand_(*k*). A *ϕ*_norm_(*k*) > 1 indicates that the rich-club phenomenon exists in the network. A *ϕ*_norm_(*k*) < 1 indicates that “rich” nodes do not tend to form a dense clique among themselves.

### Coreness properties

The coreness properties refer to a class of node properties measuring the richness of its connections and of its neighbors [27]. Eigenvector centrality and PageRank centrality are instances that fall into this category. For a network *G* = (*V*,*E*) and **A** = (*a*_*i*,*j*_) the adjacency matrix, *a*_*i*,*j*_ = 1 if there is a directed edge pointing from node *j* to node *i*. The eigenvector centrality *E*_*i*_ of node *i* is defined as the solution of

$$\begin{array}{c} E_{i}=x_{i}=\frac{1}{\lambda }\sum_{j}a_{i,j}x_{j}, \end{array}$$ (1)

where *λ* is a constant. The values **x** in *E* can be solved approximately by setting the initial values **x**(0) = 1 and repeatedly updating the values by

$$\begin{array}{c} x\left(t\right)=A^{t}x\left(0\right), \end{array}$$ (2)

for a limited number of *t* times.

PageRank centrality is an extension of eigenvector centrality with a scaling factor. The PageRank centrality *P*_*i*_ of node *i* is defined as the solution of

$$\begin{array}{c} P_{i}=x_{i}=\alpha \sum_{j}a_{i,j}\frac{x_{j}}{L_{j}}+\frac{1-\alpha }{N}, \end{array}$$ (3)

where *L*_*j*_ = ∑_*i*_ *a*_*j*,*i*_ is the in-degree of node *j* and *N* is the number of nodes in the network. In this work, we set *α* = 0.85. Note that eigenvector centrality and PageRank centrality measure the centrality of a node by the density of the inbound connections to this node and its neighbors. Therefore, larger eigenvector and PageRank centralities indicate stronger attraction of a club to football players.

### Brokerage properties

Network brokerage is used to describe the ability of a node to connect other nodes in the network. In this paper, we have chosen effective size, closeness centrality and betweenness centrality as measures of both local and global brokerage power of a node in the network. The effective size is defined by

$$\begin{array}{c} S_{i}=1-\frac{k_{i}-1}{k_{i}}C_{i}, \end{array}$$ (4)

where *k*_*i*_ is the degree of node *i* and *C*_*i*_ is its clustering coefficient [28]. *S*_*i*_ ranges between 0 and 1. It takes its smallest value when node *i* is part of a fully connected structure (hence, has no local brokerage power), and it takes its largest value when the neighbors of the node have no mutual connections (hence, the node achieves maximum local brokerage power).

Closeness centrality *Cl*_*i*_ is the reciprocal of the sum of geodesic distances to all other nodes

$$\begin{array}{c} Cl_{i}=\frac{1}{\sum {}_{j\neq i\;}d_{ij}}, \end{array}$$ (5)

where *d*_*ij*_ is the geodesic distance pointing from nodes *i* to *j*. A large closeness centrality means that the node is close to all other nodes in the network, hence can easier act as a broker from a global perspective.

The betweenness centrality *B*_*i*_ of node *i* is defined by

$$\begin{array}{c} B_{i}=\sum_{s\neq i,s\neq t,i\neq t}\frac{g_{sti}}{g_{st}}, \end{array}$$ (6)

where *g*_*st*_ is the number of directed shortest paths from nodes *s* to *t*, and *g*_*sti*_ is the number of shortest paths between nodes *s* and *t* that pass through node *i*. A large betweenness centrality can also indicate large global brokerage power.

### Kendall’s Tau

In this paper, the interrelations between network properties and club functionalities associated with the nodes in the global football player transfer network are studied. As shown in Fig 5, the distributions of the club functionality measurements are largely different from each other. Therefore, we have chosen Kendall’s Tau coefficient, which is an accepted non-parametric measure of concordance particularly suitable for detecting ordinal association between two measured quantities without assumptions of underlying distributions. Let (*x*_1_,*y*_1_), (*x*_2_,*y*_2_), …,(*x*_*n*_,*y*_*n*_) be a sequence of measurements to compare. Any pair of measurements (*x*_*i*_,*y*_*i*_) and (*x*_*j*_,*y*_*j*_), where *i* ≠ *j*, is said to be concordant if their ranks in the two sequences have the same order, i.e., if both *x*_*i*_ > *x*_*j*_ and *y*_*i*_ > *y*_*j*_ or if both *x*_*i*_ < *x*_*j*_ and *y*_*i*_ < *y*_*j*_. They are said to be discordant, if *x*_*i*_ > *x*_*j*_ and *y*_*i*_ < *y*_*j*_ or if *x*_*i*_ < *x*_*j*_ and *y*_*i*_ > *y*_*j*_. If *x*_*i*_ = *x*_*j*_ or *y*_*i*_ = *y*_*j*_, the pair is neither concordant nor discordant. Kendall’s Tau is defined as:

$$\begin{array}{c} \tau =\frac{n_{+}-n_{-}}{1/2n(n-1)}, \end{array}$$ (7)

where *n*_+_ is the number of concordant pairs and *n*_ − _ the number of disconcordant pairs. The coefficient *τ* ranges between 1 and -1, where a larger positive value indicates a stronger positive association, a larger negative value indicates a stronger negative association and a value closer to 0 indicates that the two measured quantities are weakly interrelated or independent.

## Supporting Information

S1 File This file contains Tables A–E. (PDF)

S1 Fig Scatter plot of annual balances and average league points of the clubs. (EPS)
