# Supplementary material for: The Anatomy of the Global Football Player Transfer Network: Club Functionalities versus Network Properties
Source: PLoS One. 2016 Jun 2;11(6):e0156504. doi: 10.1371/journal.pone.0156504 (PMC4890771; doi:10.1371/journal.pone.0156504)
Supplement: S1 File — (PDF) [file pone.0156504.s001.pdf]

Table A. Top 10 clubs in terms of the annual expense, revenue and volume in the transfer market.

Rank	Annual Revenue	Annual Expense	Annual Volume
1	FC Porto (Portugal)	Chelsea FC (England)	Chelsea FC (England)
2	Benfica (Portugal)	Manchester City FC (England)	Liverpool FC (England)
3	Tottenham Hotspur FC (England)	Paris Saint Germain FC (France)	Manchester City FC (England)
4	Club Atletico de Madrid (Spain)	Manchester United FC (England)	Real Madrid CF (Spain)
5	Liverpool FC (England)	Liverpool FC (England)	Manchester United FC (England)
6	Chelsea FC (England)	Real Madrid CF (Spain)	Club Atletico de Madrid (Spain)
7	Real Madrid CF (Spain)	FC Barcelona (Spain)	Tottenham Hotspur FC (England)
8	AS Monaco FC (Monaco)	Juventus FC (Italy)	Paris Saint Germain FC (France)
9	AS Roma (Italy)	Club Atletico de Madrid (Spain)	AS Monaco FC (Monaco)
10	Valencia CF (Spain)	FC Bayern Munchen (Germany)	FC Barcelona (Spain)

Table B. Top 10 clubs in terms of the match performances.

Rank	Average League Points	Aggregate CWR Points
1	Olympiakos CFP (Greece)	FC Barcelona (Spain)
2	FC shakhtar Donetsk (Ukraine)	Real Madrid CF (Spain)
3	FC Bayern Munchen (Germany)	FC Bayern Munchen (Germany)
4	FC Barcelona (Spain)	Club Atletico de Madrid (Spain)
5	Benfica (Portugal)	Paris Saint Germain FC (France)
6	Celtic FC (Scotland)	SSC Napoli (Italy)
7	FC Porto (Portugal)	Benfica (Portugal)
8	Real Madrid CF (Spain)	BV Borussia 09 Dortmund (Germany)
9	Juventus FC (Italy)	Manchester City FC (England)
10	FC Dynamo Kyiv (Ukraine)	Celtic FC (Scotland)

Table C. Top 10 clubs in terms of the profitability in the transfer market.

Rank	Balance	Price overflow
1	FC Porto (Portugal)	AS Monaco FC (Monaco)
2	Benfica (Portugal)	FC Porto (Portugal)
3	Lille Olympique Sporting Club Metropole (France)	Benfica (Portugal)
4	Santos FC Sao Paulo (Brazil)	AS Roma (Italy)
5	Sevilla FC (Spain)	VFL Wolfsburg (Germany)
6	AFC Ajax (Netherlands)	Torino FC (Italy)
7	Udinese Calcio (Italy)	Bayer 04 Leverkusen (Germany)
8	US Citta di Palermo (Italy)	Club Atletico de Madrid (Spain)
9	Royal Sporting Club Anderlecht (Belgium)	Sevilla FC (Spain)
10	Olympique Lyonnais (France)	Newcastle United FC (England)

Table D. Top 10 clubs in terms of the coreness properties in the player transfer network.

Rank	Eigenvector centrality	PageRank centrality
1	Club Atletico de Madrid (Spain)	Toronto FC (Canada)
2	Sporting Clube de Portugal (Portugal)	CS Concordia Chiajna (Romania)
3	Juventus FC (Italy)	Chicago Fire (United States)
4	FC Internazionale Milano (Italy)	New York Red Bulls (United States)
5	Benfica (Portugal)	Sporting Clube de Portugal (Portugal)
6	AC Milan (Italy)	Udinese Calcio (Italy)
7	AS Roma (Italy)	FC Porto (Portugal)
8	ACF Fiorentina (Italy)	Olympiakos CFP (Greece)
9	Genoa CFC (Italy)	KV Red Star Waasland (Belgium)
10	Sevilla FC (Spain)	Club Brugge KV (Belgium)

Table E. Top 10 clubs in terms of the brokerage properties in the player transfer network.

Rank	Effective size	Betweenness centrality	Closeness centrality
1	Sporting Clube de Portugal (Portugal)	Sporting Clube de Portugal (Portugal)	HIFK Soccer Helsinki (Finland)
2	Benfica (Portugal)	Helsingin Jalkapallokubi (Finland)	FC Internazionale Milano (Italy)
3	Sevilla FC (Spain)	FC Porto (Portugal)	Sporting Clube de Portugal (Portugal)
4	FC Porto (Portugal)	Chicago Fire (United States)	AFC Ajax (Netherlands)
5	AS Roma (Italy)	Sevilla FC (Spain)	Club Atletico de Madrid (Spain)
6	Club Atletico de Madrid (Spain)	Olympiakos CFP (Greece)	PSV NV (Netherlands)
7	Olympiakos CFP (Greece)	SCS CFR 1907 Cluj SA (Romania)	Sevilla FC (Spain)
8	Sporting Braga (Portugal)	Club Brugge KV (Belgium)	FC Porto (Portugal)
9	AS Monaco FC (Monaco)	Standard de Liege (Belgium)	FC Barcelona (Spain)
10	FC Internazionale Milano (Italy)	Sporting Braga (Portugal)	Benfica (Portugal)
